# Prospects for online adaptive radiation therapy (ART) for head and neck cancer

**DOI:** 10.1186/s13014-023-02390-6

**Published:** 2024-01-08

**Authors:** Maja Guberina, Nika Guberina, C. Hoffmann, A. Gogishvili, F. Freisleben, A. Herz, J. Hlouschek, T. Gauler, S. Lang, K. Stähr, B. Höing, C. Pöttgen, F. Indenkämpen, A. Santiago, A. Khouya, S. Mattheis, M. Stuschke

**Affiliations:** 1 Department of Radiotherapy, West German Cancer Center, University Hospital Essen, Hufelandstraße 55, 45147 Essen, Germany; 2 Department of Otorhinolaryngology, Head and Neck Surgery, University Hospital Essen, Essen, Germany; 3 Present Address: German Cancer Consortium (DKTK), Partner Site University Hospital Essen, Essen, Germany

**Keywords:** Online adaptive radiation therapy, Head and neck, Organs at risk, Automatic segmentation, Dose gradients

## Abstract

**Background:**

The aim of the present study is to examine the impact of kV-CBCT-based online adaptive radiation therapy (ART) on dosimetric parameters in comparison to image-guided-radiotherapy (IGRT) in consecutive patients with tumors in the head and neck region from a prospective registry.

**Methods:**

The study comprises all consecutive patients with tumors in the head and neck area who were treated with kV-CBCT-based online ART or IGRT-modus at the linear-accelerator ETHOS™. As a measure of effectiveness, the equivalent-uniform-dose was calculated for the CTV (EUD_CTV_) and organs-at-risk (EUD_OAR_) and normalized to the prescribed dose. As an important determinant for the need of ART the interfractional shifts of anatomic landmarks related to the tongue were analyzed and compared to the intrafractional shifts. The latter determine the performance of the adapted dose distribution on the verification CBCT2 postadaptation.

**Results:**

Altogether 59 consecutive patients with tumors in the head-and-neck-area were treated from 01.12.2021 to 31.01.2023. Ten of all 59 patients (10/59; 16.9%) received at least one phase within a treatment course with ART. Of 46 fractions in the adaptive mode, irradiation was conducted in 65.2% of fractions with the adaptive-plan, the scheduled-plan in the remaining. The dispersion of the distributions of EUD_CTV_-values from the 46 dose fractions differed significantly between the scheduled and adaptive plans (Ansari-Bradley-Test, *p* = 0.0158). Thus, the 2.5th percentile of the EUD_CTV_-values by the adaptive plans amounted 97.1% (95% CI 96.6–99.5%) and by the scheduled plans 78.1% (95% CI 61.8–88.7%). While the EUD_CTV_ for the accumulated dose distributions stayed above 95% at PTV-margins of ≥ 3 mm for all 8 analyzed treatment phases the scheduled plans did for margins ≥ 5 mm. The intrafractional anatomic shifts of all 8 measured anatomic landmarks were smaller than the interfractional with overall median values of 8.5 mm and 5.5 mm (*p* < 0.0001 for five and *p* < 0.05 for all parameters, pairwise comparisons, signed-rank-test). The EUD_OAR_-values for the larynx and the parotid gland were significantly lower for the adaptive compared with the scheduled plans (Wilcoxon-test, *p* < 0.001).

**Conclusions:**

The mobile tongue and tongue base showed considerable interfractional variations. While PTV-margins of 5 mm were sufficient for IGRT, ART showed the potential of decreasing PTV-margins and spare dose to the organs-at-risk.

**Supplementary Information:**

The online version contains supplementary material available at 10.1186/s13014-023-02390-6.

## Introduction

Radiation therapy and concurrent chemotherapy is the curative treatment option for inoperable, locally advanced tumors in the head and neck area and of high effectiveness in the postoperative situation in the presence of high-risk features (R1 and/or nodal ECE+) [1, 2]. Anatomic changes can occur despite efforts to achieve reproducible positioning from fraction to fraction. Radiochemotherapy-sensitive tumors shrink under therapy. Likewise, the patient may lose weight under combined radiochemotherapy. These systematic changes make an early plan adaptation during a treatment phase necessary [3–6]. However, systematic changes are not the only issue, which may lead to tumor deviation. Internal motion of the tongue and soft tissue in the head and neck area during a treatment session may further add to deviations. The frequency and duration of deglutition varies interindividually and with time [7]. Deviations in the head and neck area may occur even when the patient does not swallow due to respiration and tongue movement [7]. Nevertheless, accounting for all possible motion pathways inevitably leads to large PTV margins in an area that is complex and where many critical organs are at risk in a confined space. Many previous groups showed that accurate image guidance is mandatory to deposit a sufficient dose to the gross tumor volume (GTV) and clinical target volume (CTV) by minimizing toxicity to organs at risk [8, 9]. Excellent image guidance and techniques such as intensity modulated radiotherapy (IMRT) and volumetric modulated arc therapy (VMAT) are necessary for high precision radiation therapy using steep dose gradients towards organs at risk. Anatomic deviations can affect both the dose coverage of the CTV as well as dose distribution to normal tissues as parotid gland, oral cavity and spinal cord depending on which region of interest the rigid registration using IGRT is focused on [9]. Despite advanced imaging techniques radiation dose to organs at risk may exceed a predefined and intended limit [10].

Adaptive radiation therapy is the latest development, which promises to combine high precision therapy and the possibility to reduce treatment margins in the head and neck area. Retrospective studies on offline adaptive radiation therapy (ART_offline_) for head and neck cancer showed that re-planning with adaptive radiotherapy may ensure adequate dose coverage and sparing of organs at risk [11–13]. In a virtual retrospective analysis, Franzese et al. simulated the relevance of an adaptive strategy for head–neck cancer patients treated with definitive or post-operative radiotherapy [11]. Their results showed that in the absence of re-planning doses to the analyzed organs at risk may increase during the long course of radiotherapy delivered with the VMAT technique with a potential clinical impact in terms of increased toxicity [11]. Likewise, Liu et al. performed a retrospective planning study using treatment plans for four different treatment strategies, including a solely image guided radiation therapy (IGRT) strategy (IGRT-only), two adaptive treatment planning strategies using 3- and 0-mm planning target volume (PTV) margins, and the 4D ART_offline_ strategy [14]. The authors conclude that the use of 4D ART_offline_ improved target coverage and attained OAR sparing similar to that with 0-mm ART [14–18].

Mahmoud et al. [15] concluded that ART_offline_ is important particularly in patients with bulky head and neck cancer due to target under dosing and/or spinal cord/parotids overdosing in weeks 3 and 6. Reasons for these dosimetric alterations were patient weight loss, which was almost twice as high in the definitive compared to the postoperative group. Furthermore, tumor shrinkage was another reason leading to alterations, which made adaptive replanning important. Several authors tried to find offline the best point during treatment, when to switch to ART [13, 19–23]. However, a randomized phase-3-trial on weekly offline adaptation showed a rather sober outcome and no benefit for reducing xerostomia in oropharyngeal cancer [24].

A more close-to-patient approach is online adaptive radiation therapy. It allows the instant online planning within one session [25]. Contrary to retrospective studies on ART or offline ART, this allows to react instantly to CTV deformations onboard during online navigation. Currently, linear accelerators which allow online adaptive re-planning are coming on the market [26, 27]. In addition to MR-linacs, online adaptive radiation therapy (ART) is made possible by replanning on daily anatomy captured on a high-quality cone-beam computed tomography (CBCT) [26].

Hence, the aim of the present study is to examine the value of kV-CBCT-based online ART_online_ in the first clinical setting of patients with tumors in the head and neck area using the online adaptive mode (subsequently for better readability just referred to as ART). In detail, the recalculated dose distribution visualized on the daily CBCT with the structures deformed onto the CBCT and the value of these options for performing onboard navigation will be evaluated in the first, consecutive, real life patient cohort with tumors in the head and neck area.

## Material and methods

### Patient cohort

The study comprises all consecutive patients with tumors in the head and neck area who were treated at the linear accelerator ETHOS (Varian, Palo Alto, US) at the Department of Radiation Therapy of University Hospital Essen in the time period from 01.12.2021 to 31.01.2023. All fractions of patients who received radiation therapy in the adaptive mode were assessed. Adaptive radiotherapy (ART) was conducted under the discretion of an expert radiation oncologist. ART was chosen especially for tumors located near sensitive organs at risk. Other indications were the critical need for tissue sparing, e.g. limiting the dose to the oral cavity. Main exclusion criteria were tumor infiltration of the skin or the necessity of using bolus material. All treated patients gave their consent to the treatment taking part in the prospective, institutional clinical registry trial (18-8364-BO). The study was conducted in accordance with the principles of the Declaration of Helsinki. The study was approved by the Ethics committee of University Hospital Essen of University of Duisburg-Essen (21-10465-BO).

### Treatment planning

According to present international guidelines [29] patient cases were discussed in an interdisciplinary tumor board as part of the West German Cancer Center and the National Center for Tumor Diseases (NCT). Patients were seen by an expert radiation oncologist, who clarified all treatment modalities, risks and alternatives. The planning CT was acquired with contrast agent for a better tumor delineation. Treatment was delivered in plain free breathing once daily with a homogenous fractionation dose of 5*2 Gy/w (q.e.d.) to a total prescription dose of 64–66 Gy for postoperative and of 70 Gy to the macroscopic tumor for definitive treatments with 32–35 fractions as a continuous course using two or three sequential treatment phases. The first phase was delivered to the lower-risk targets, such as nodal neck levels which are not first echelon nodes. The second phase, if used, was delivered to the high-risk subclinical disease sites. These involve anatomical compartments containing the GTV or the preoperative GTV with a 10 mm CTV-margin and first echelon nodes, which are not clinically or radiologically involved. The third phase comprises GTV or the preoperative GTV with a 5 mm clinical target volume margin not crossing anatomic borders [30–32]. Elective lymph nodes regions at risk were delineated according to the EORTC consensus [33, 34]. To guarantee constant positioning a thermoplastic mask system was always used. The treatment planning system ECLIPSE (Varian. Palo Alto. US) was used for treatment preparation. Organs at risk (OAR), gross tumor volume (GTV) and clinical target volume (CTV) were defined by fusing computed tomography and magnetic resonance imaging with the planning CT following present contouring consensus guidelines [30]. The treatment plan was calculated either as volumetric modulated arc therapy (VMAT) or static-field intensity modulated radiotherapy (IMRT) plan. The planning target volume (PTV)-margins were 3.0–5.0 mm to consider potential set-up errors. Using the ETHOS-integrated dose calculation (version 1.1.2.44. primary fluence mode FFF, 6 MV) important dose descriptors for the deformed CTV_ij_ of each fraction i and patient j were calculated and compared between (1) reference (original plan on anatomy of planning CT), (2) scheduled (reference plan on anatomy of the day) and (3) adaptive plans (adaptive plan on anatomy of the day).

### Adaptive radiotherapy (ART) and treatment modes

In the adaptive mode, the system displays an adaptive dose distribution and a planned dose distribution for each fraction between which the more appropriate plan for the treatment must be selected. In contrast to the pure IGRT mode, which only allows adjustments for the degrees of freedom implemented in the treatment couch, ART allows both IGRT as an integral part of any modern radiotherapy sequence and online plan adjustment. After having optimized the adaptive plan, there is a choice to treat with the initial scheduled plan in the IGRT mode or with the adaptive plan according to the decision of the treating radiation oncologist together with the medical physicist.

The BODY contour is an important factor in head and neck planning. The upper airways and externa such as bite block or thermoplastic mask systems may pose a severe challenge for correct identification of the BODY contour by the ETHOS system. An incorrect BODY contour inevitably leads to wrong dose calculation and failure of the planning procedure. However, this structure must be correctly defined within the adaptive ETHOS system. This can be done by manually defining this structure externally in advance, e.g. in the Varian Eclipse system, importing it into the ETHOS software, and then locking this BODY structure with the LOCK key. By locking the body, it is achieved that no high-level deviation of the BODY structure may occur, and it is continuously defined correctly with minor deviation.

During an adaptive session, influencers (organs at risk that influence deformation of targets) are segmented by an intensity-based deformation algorithm. Prior to the next step, these contours have to be checked and accepted by an expert radiation oncologist. In cases in which OAR and influencer contours need a fine-tuning, the treating physician makes edits based on a high-quality CBCT, which allows the next step of the adaptive workflow. Subsequently, clinical target volumes are deformed to the CBCT and once again, these structures have to be edited and approved by the treating radiation oncologist. Finally, an adaptive plan is automatically calculated on a synthetic CT based on the original plan with regard to the present expert-supervised, segmented onboard morphology. The adaptive plan is created with planning parameters and constraints as the original plan. For every fraction, the system shows an adaptive dose distribution and a scheduled dose distribution, which represents the original plan calculated on the daily anatomy. The adaptive plan is compared with the original and the scheduled plan by the treating expert radiation oncologist and medical physicist. The mean doses to normal tissues, as the parotid glands, the mandible and larynx, as well as maximum doses to the spinal cord were compared. After plan approval, a second low-dose CBCT 2 is acquired in order to verify positioning in an IGRT mode. The minimum required margin to achieve an accumulated EUD_CTV_ > 95% for a treatment phase containing ≥ 3 dose fractions was retrospectively offline determined by stepwise creating synthetic CTV_1–5 mm_ volumes from a given PTV by shrinking the PTV using inner margins of 1–5 mm in the planning CT and calculating the EUD for the resulting CTV_1mm-5 mm_ under the accumulated dose distribution.

### Primary and secondary endpoints

The primary endpoint is to examine the added value of online adaptive radiotherapy in terms of equivalent uniform dose (EUD) of the clinical target volume and organs at risk (OAR). The EUD quantifies the effect of a non-homogeneously dose distribution [35]. The EUD is determined according to the phenomenological power law model described by [36]. The equivalent uniform dose is calculated giving the same biological effect on the tumor. For tumors we use the tissue specific parameter a =  − 20, that is appropriate for aggressive tumors [37]. Given these exponents makes the EUD sensitive to dose minima [38]. For the parotid gland a value of a = 1.43 and the larynx of 9.1 is used [39]. Throughout this study, all EUD values are normalized to the prescribed dose. Secondary endpoints are to examine important dose metrics for the clinical target volume (1) V100 (the volume which receives 100% of prescribed dose), (2) D_max_ (the maximum dose within the CTV), (3) D99 (the dose that irradiates 99% of the CTV), (4) D95 (the dose that irradiates 95% of the CTV), (5) D90 (the dose that irradiates 90% of the CTV), and (6) D_min_ (the minimum dose within the CTV). In order to account for acute toxicity all patients were checked for therapy related tissue changes weekly. Toxicity was also examined 6–8 weeks after completing therapy as part of routine follow-up. The EORTC/RTOG scoring system to evaluate acute toxicity and the Fox Chase (FC) modification of RTOG and the Late Effects Normal Tissue Task Force (LENT) scoring system were applied to examine late toxicity after treatment completion.

### Inter- and intrafractional movement

ART is particularly important for radiotherapy with large interfractional deformations of the CTV, which has to be compensated with planning target volume margins. ART may allow smaller PTV volumes only in case intrafractional deviations are smaller than interfractional. Hence, we examined interfractional deviations between planning-CT and CBCT1 as well as intrafractional movement between CBCT 1 and CBCT 2. Inter- and intrafractional deformations were evaluated by measuring specific points at predefined landmarks on the sagittal plane in the midline of the planning-CT. CBCT1 and CBCT2: (a) anterior/posterior deviation of posterior pharyngeal wall at the tip of the epiglottis; (b) maximum anterior/posterior deviation of posterior pharyngeal wall; (c) maximum deviation of mandible; (d) maximum craniocaudal deviation of os hyoideum; (e) overall maximum deviation of os hyoideum; (f) maximum anterior/ posterior deviation of tongue base; (g) maximum craniocaudal deviation of tongue back; and (h) anterior/ posterior deviation of tongue base at the tip of the epiglottis (Additional file 1: Fig. S1).

### Statistics

Descriptive and statistical analysis was performed with IBM SPSS Statistics version 29.0 and SAS statistical software system SAS/STAT 15.1 (SAS Institute Inc. Cary NC. USA). Differences in location between the cumulative distribution functions of two independent distributions, e.g. the position of a landmark in the initial pre-adaptation CT and the verification CT after dose adaptation from the same dose fraction, were compared by the Wilcoxon-test. Differences in scale, the dispersion of the data of distributions around their median, were analyzed by the Mood-test (procedure napar1way, SAS). Differences in locations of two distributions of paired values, e.g. the interfractional deviation of an internal anatomic landmark in the initial CBCT1 from that in the planning CT, and the intrafractional deviation of the same landmark in CBCT2 from that in CBCT1 from the same dose fraction from 0, were analyzed by Wilcoxon signed-rank test. Pearson-correlation was used to assess possible dependencies between important dose metrics. A 2-sided *p* value of < 0.05 was considered as significant. Additionally, a multivariate correlation was performed to examine a possible dependence of anatomical deformations on EUD-values.

## Results

In the time period from 01.12.2021 to 31.01.2023 59 patients with tumors in the head and neck area were treated at the ETHOS system of the Department of Radiation Therapy of University Hospital Essen. Altogether ten of all 59 patients (10/59; 16.9%) received at least one phase within a course with the adaptive mode under the discretion of an expert radiation oncologist who was involved in the treatment planning. ART was mainly used for tumors of the tongue base and mobile tongue. A larger proportion of the fractions that were carried out in ART mode were carried out within the boost course (70% of all cases). Table 1 summarizes patient characteristics treated with the adaptive mode. Altogether 30/46 fractions in the adaptive mode were delivered with the adaptive plan (65.2% applied adaptive fractions).

**Table 1:** Delineation of characteristics of all consecutive patients with tumors in the head and neck area who were treated at the linear accelerator ETHOS™ at the Department of Radiation Therapy of University Hospital Essen in the time period from 01.12.2021 to 31.01.2023

	IGRT	ART for boost	ART for initial course
*Definitive RT/CTX*			
Oropharyngeal	12	1	0
Oral cavity	13	1	0
Hypopharyngeal	0	0	0
Larynx	2	0	0
CUP syndrome	0	0	0
Other	1	0	0
Stage III/IVA/IVB	25	0	0
P16 positive	6	1	0
*Postoperative RT/CTX*			
Oropharyngeal	10	4	2
Oral cavity	7	1	1
Hypopharyngeal	1	0	0
Larynx	1	0	0
CUP syndrome	0	0	0
Other	2	0	0
Stage III/IVA/IVB	19	3	3
P16 positive	7	2	1

Numbers indicating the number of patients in each category. All 59 patients were treated within the IGRT mode. 10/59 patients received additionally a phase within the initial or the boost course with the ART mode

The adaptive treatment lasted in median 34.5 min (range: 12.0–49.0 min) from opening the patient online file, patient positioning onboard, CBCT-acquisition, structure segmentation, plan generation and review, second CBCT-acquisition and treatment delivery. Propagated, segmented CTV_i_s required none or minor editing in 44.2% and 48.0%. Altogether 7.7% required intermediate or major editing of propagated CTV_i_s.

**Fig. 1:**
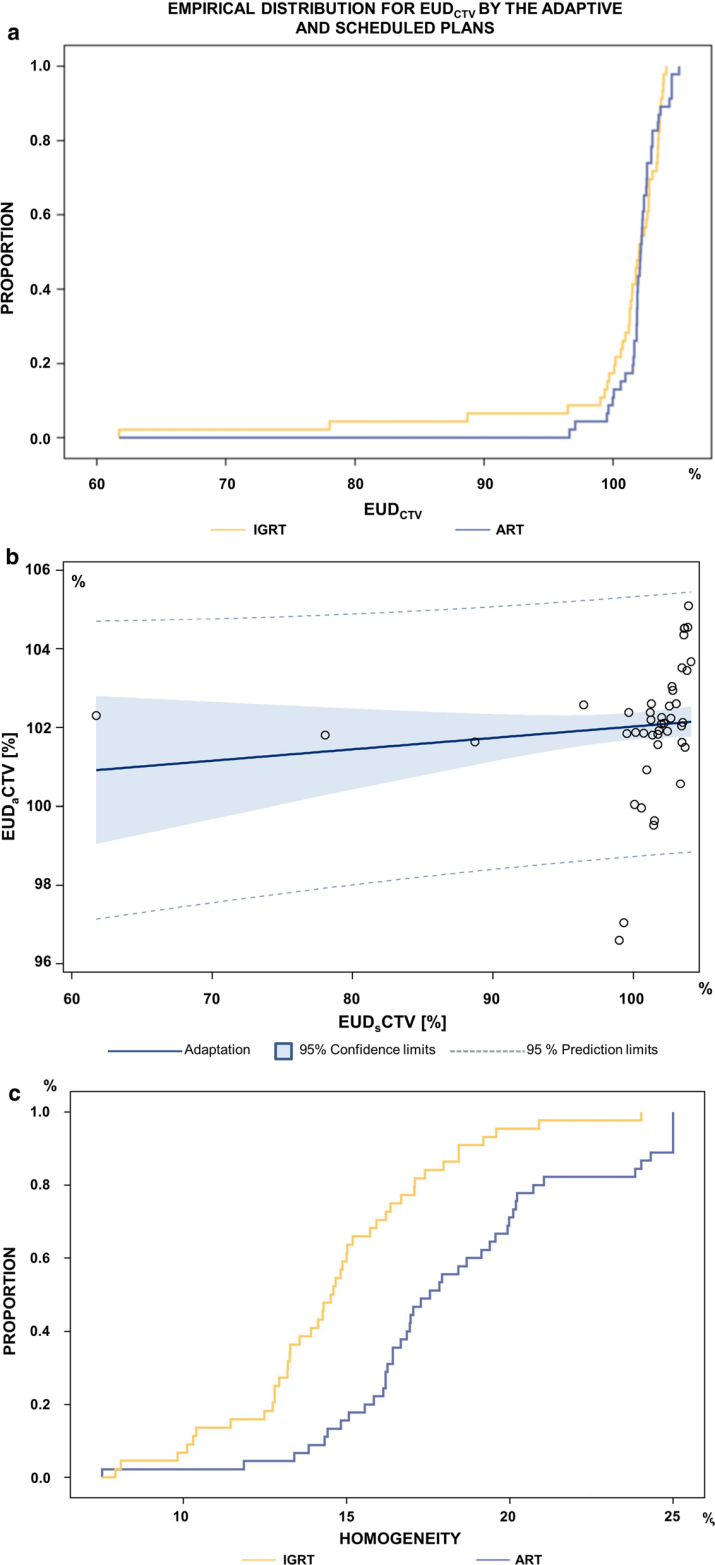
**a** Empirical distribution functions of the EUD_CTV_ values per dose fraction by the adaptive and scheduled plans. **b** Adaptation plot of EUDaCTV (EUD within the CTV in the adaptive plan). EUDaCTV remained at about 100% of the planned EUD for the DIR-based accumulated dose distributions independent from the EUDsCTV, implying that predominantly patients with poor EUD in the scheduled plan benefited from adaptation (*p* = 0.11). The intercept was 99.5% ± 3.5%, the slope was 0.024% ± 0.035% (*p* = 0.5). **c** Empirical distribution functions of the dose homogeneity by the adaptive and scheduled plans. Dose homogeneity of the dose distributions within the CTV was significantly better for the adaptive plans. The empirical distribution functions of the differences D_max_CTV_ − D_99_CTV_ as a measure of dose homogeneity with smaller differences found for the adaptive in comparison to the scheduled plans (*p* < 0.0001, signed rank test for paired differences between homogeneity measures from the same fraction)

The median EUD_CTV_ per dose fraction for the clinical target volume by the scheduled plans in CBCT 1 was 102.0% (range 61.8–104.1%); the median EUD for the clinical target volume in all adaptive plans (CTV_adaptive_) in CBCT 1 was 102.1% (range 96.6–105.1%). While the median of the EUD_CTV_ values by all adaptive plans did not significantly differ from the EUD_CTV_ values from the scheduled plans (*p* = 0.5491, Wilcoxon-signed-rank-test), the scale of the EUD_CTV_-distributions as a measure of dispersion, however, was significantly different (Ansari-Bradley Test, *p* = 0.0158). Especially, in the low dose tail of the distributions, the 2.5th percentile of the EUD_CTV_ values by the adaptive plan amounted 97.1% (95% CI 96.6–99.5%) and by the scheduled plan 78.1% (95% CI 61.8–88.7%). A EUD_CTV_ decline by the scheduled plan was not correlated with a decline by the adaptive plan (r = 0.1255, *p* = 0.41). The empirical distribution functions of the EUD_CTV_ values by the adaptive and scheduled plans are shown in Fig. 1a.

EUD_CTV_-values for the adaptive plan remained at around 100% of the prescribed dose independent from those in the scheduled plan, implying that predominantly patients with poor EUD_CTV_ in the scheduled plan benefited from adaptation.

Eight treatment phases for different patients contained three or more dose fractions, amounting in total 43 fractions from this study. The EUD_CTV_ values for the DIR-based, accumulated dose distributions per phase by the adaptive plans versus those by the scheduled plans are shown in Fig. 1b.

Median D_99%_ for CTV by the scheduled plan was 96.7% (range 69.4–99.2%) compared to median values of 97.8% (range 91.7–99.1%) for CTV_ij_ by the adaptive fractions. Likewise, D_min_CTV_ for CTV within the scheduled plan was significantly lower with median values of 81.3% (range 37.6–97.0%) compared to median values of 88.8% (range 52.8–96.6%) for CTV in the adaptive fractions. D_min_ was the most important metrics correlated with the EUD_CTV_ (Pearson-correlation *p* < 0.01) for both scheduled and adaptive plan in either adaptive applied as well as adaptive non-applied fractions. In addition, homogeneity of the dose distributions within the CTV was significantly higher for the adaptive plans. Figure 1c shows the empirical distribution functions of the differences D_max_CTV_ − D_99_CTV_ as a measure of dose homogeneity with smaller differences found for the adaptive in comparison to the scheduled plans (*p* < 0.0001, signed rank test for paired differences between homogeneity measures from the same fraction).

In each case, the intrafractional deviations by the corresponding parameters between CBCT1 and CBCT2 were evaluated. The interfractional deviations were assessed by the corresponding parameters between CBCT1 and planning CT. The difference of the absolute interfractional and the absolute intrafractional deviation was calculated (delta). Delta for all parameters was significantly positive (signed rank test), i.e. interfractional variability is larger than intrafractional. 6 of 8 parameters associated with interfractional anatomical deviation of the tongue showed significant deviations of more than 5 mm with respect to the 95th percentile and thus a high mobility despite an individualized bite block.

**Fig. 2:**
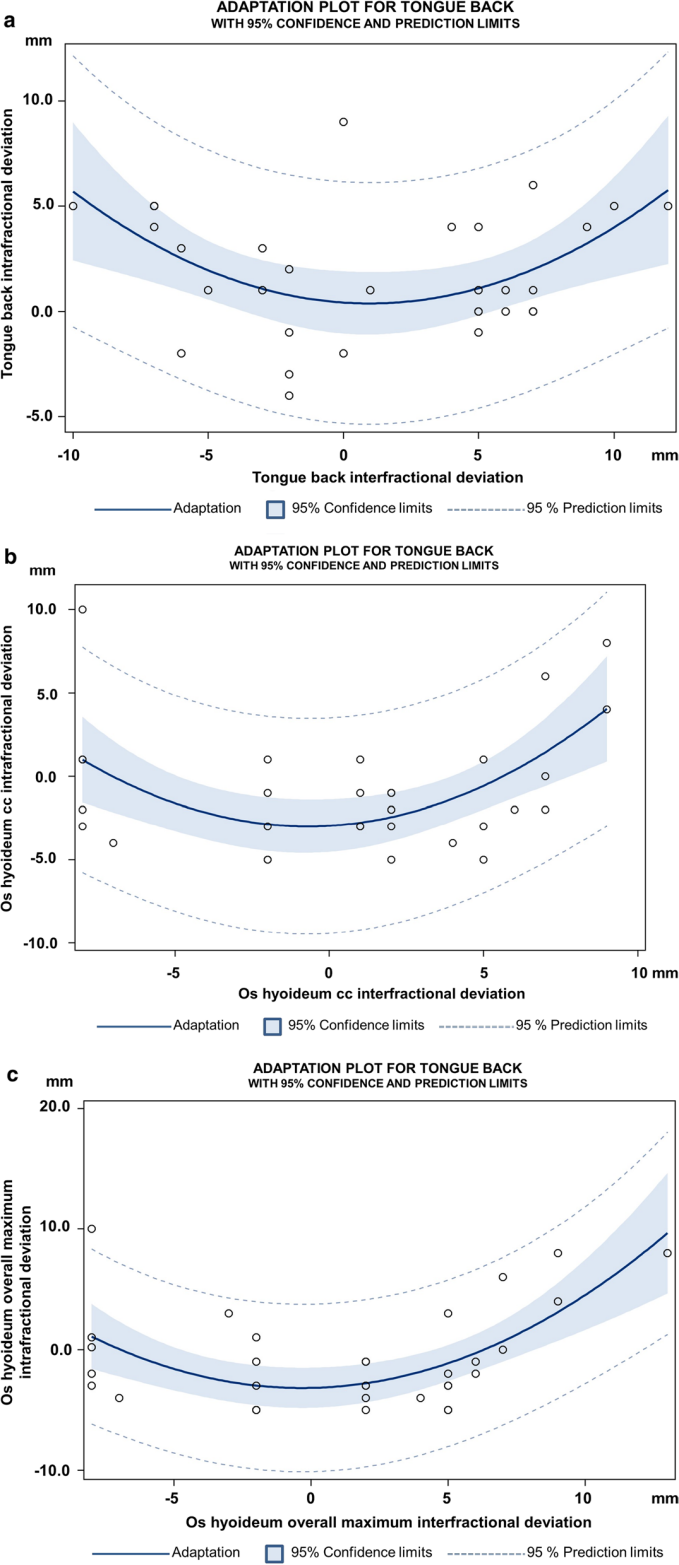
**a** Adaptation plot for tongue back. The intrafractional variability of tongue back was significantly correlated with the interfractional variability, which means that intrafractional deviations may be larger for large interfractional deviations (*p* = 0.0076). **b** Adaptation plot for os hyoideum. The intrafractional variability of maximum cranicocaudal deviation of the os hyoideum was significantly correlated with the interfractional variability, which means that intrafractional deviations may be larger for large interfractional deviations (*p* = 0.0008). **c** Adaptation plot for os hyoideum. The intrafractional variability of maximum overall deviation of the os hyoideum was significantly correlated with the interfractional variability, which means that intrafractional deviations may be larger for large interfractional deviations (*p* = 0.0002)

The intrafractional variability of examined parameters was significantly correlated with the interfractional variability, which means that intrafractional deviations may be larger for large interfractional deviations (tongue back *p* = 0.0076; os hyoideum maximum craniocaudal deviation and os hyoideum maximum overall deviation *p* = 0.0008 and *p* = 0.0002), Fig. 2a–c.

Sparing of organs at risk was systematically targeted and constraints were set as in the reference plan. Comparing EUD-values of organs at risk (parotids, larynx, spinal canal and plexus brachialis) of all fractions showed that adaptive and scheduled plans differed significantly for parotids (*p* < 0.001, two-sided exact Wilcoxon-test) and larynx (*p* < 0.001, two-sided Wilcoxon-test) with higher doses in the scheduled plan. There was no significant difference for spinal cord and plexus brachialis (0.397 and 0.143. two-sided Wilcoxon-test).

**Fig. 3:**
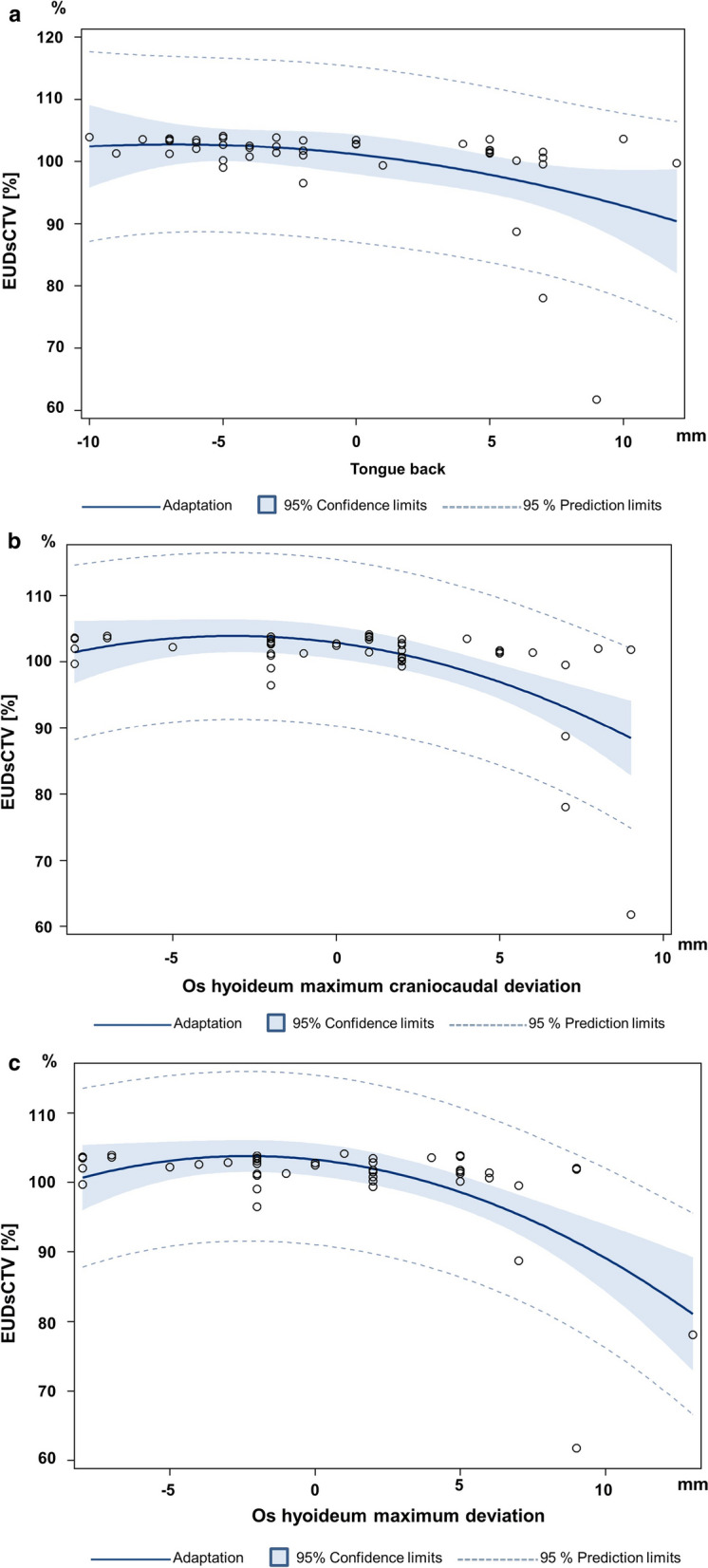
**a** Dependence of the ranks of the EUD_CTV_ by the scheduled plan (EUD_s_CTV) (1 for the highest EUD_s_CTV, 0 for the lowest EUD_s_CTV; for the 44 adaptive fractions with non-missing values) on the deviation of the tongue back in cranial (+) or caudal (−) direction between CBCT1 and the planning CT, *p* = 0.0002 [in (mm) Spearman correlation coefficient rs =  − 0.52 (95% CI − 0.71 to − 0.26)]. **b** Dependence of the ranks of the EUD_CTV_ by the scheduled plan (EUD_s_CTV) (1 for the highest EUD_s_CTV, 0 for the lowest EUD_s_CTV; for the 44 adaptive fractions with non-missing values) on the maximum craniocaudal deviation of the os hyoideum in cranial (+) or caudal (−) direction between CBCT1 and the planning CT, *p* = 0060 [in (mm) Spearman correlation coefficient rs =  − 0.40 (95% CI − 0.63 to − 0.12)]. **c** Dependence of the ranks of the EUD_CTV_ by the scheduled plan (EUD_s_CTV) (1 for the highest EUD_s_CTV, 0 for the lowest EUD_s_CTV; for the 44 adaptive fractions with non-missing values) on the maximum overall deviation of the os hyoideum in cranial (+) or caudal (−) as well as in posterior (+) or anterior (−) direction between CBCT1 and the planning CT, *p* = 0.0077 [in (mm) Spearman correlation coefficient r =  − 0.39 (95% CI − 0.62 to − 0.11)]. Highlights that the EUD_CTV_ by the scheduled plan (EUD_s_CTV) strongly depends on the deviation of the tongue back and os hyoideum. *EUD*: Equivalent Uniform Dose, *s*: scheduled plans and corresponding EUDs

Figure 3a–c highlights that in the scheduled plan the EUD_s_CTV (EUD within the CTV in the scheduled plan) strongly depends on the deviation of the tongue back and os hyoideum (maximum craniocaudal and overall maximum deviation). The coefficient of determination for the linear quadratic fit was r^2^ = 0.189, *p* = 0.0136 for the maximum anterior/ posterior deviation of the tongue back, r^2^ = 0.327, *p* = 0.0002 for the maximum craniocaudal deviation of the os hyoideum and r^2^ = 0.382, *p* < 0.0001 for the overall maximum deviation of the os hyoideum. The Spearman rank correlation coefficient between EUD_CTV_ by the scheduled plan and tongue back deviation is rs =  − 0.45 (− 0.66 to − 0.18), *p* = 0.0017, between EUD_CTV_ by the scheduled plan and maximum craniocaudal os hyoideum deviation is rs =  − 0.41 (− 0.63 to − 0.13). *p* = 0.0041, as well as between EUD_CTV_ by the scheduled plan and maximum overall hyoideum deviation rs =  − 0.39 (− 0.62 to − 0.11), *p* = 0.0077 (see Fig. 3a–c).

**Fig. 4:**
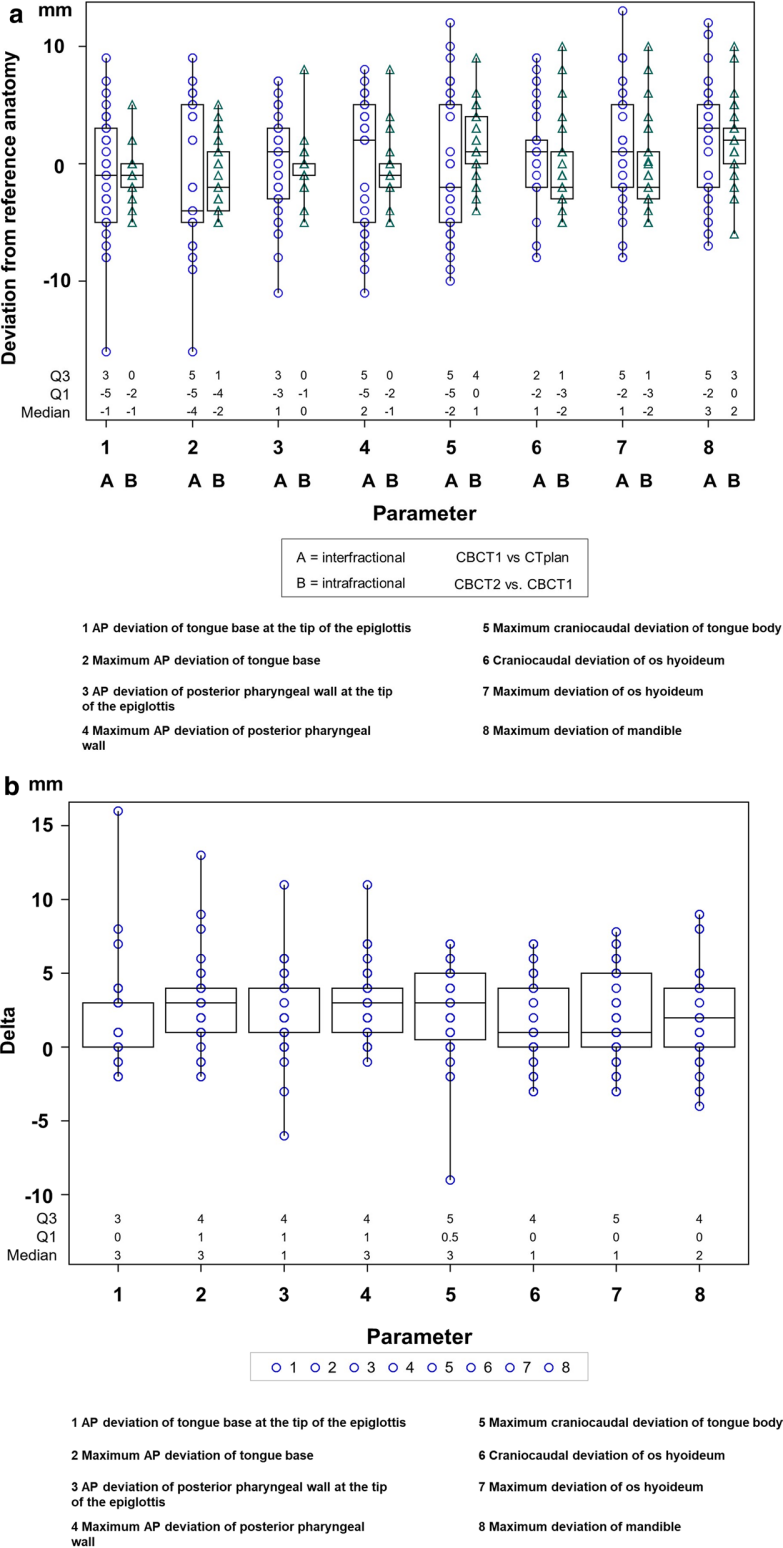
**a** Highlights the inter- and intrafractional anatomical shifts juxtaposed for parameters 1–8. The classification parameter classifies the anatomical parameters 1–8 in the order of their legend for the planning-CT versus CBCT1 (A = interfractional) and for CBCT2 versus CBCT1 (B = intrafractional). **b** Plot of difference between the absolute values of the interfractional and the intrafractional anatomical shifts for each fraction. The classification parameter classifies the anatomical parameters 1–8 in the order of their legend

Eight parameters, related to the deformation of the tongue were determined interfractional (between the CBCT1 and the planning CT) and intrafractional between (CBCT2 and CBCT1). As ART takes more than 10 min time, a gain of ART can only be expected, if intrafractional deformations are smaller than the interfractional. The location of the interfractional and intrafractional deviations, i.e. the median value does not differ significantly for any of the anatomical parameters measured at the level of alpha = 0.01 (Wilcoxon tests in each case). On the other hand, the scatter (scale) of the interfractional deviations around the median is substantially larger than the intrafractional scatter for the parameters 1, 2, 3, 4 and 5 at *p* < 0.0001 (Ansari-Bradley test) (Fig. 4a). For the other parameters the *p* values were > 0.01. The difference between the absolute values of the interfractional and the intrafractional anatomical deviations for each fraction is depicted in Fig. 4b for the 8 parameters. From these paired values, it can be found that the absolute value of the interfractional deviations per fraction was larger than the intrafractional. The median of paired difference of the absolute values was larger than 0 for parameters 1–5 at a *p* value of *p* < 0.0001 using the signed rank test, and for parameter 6, 7 and 8 at *p* values = 0.0148, 0.036 and 0.009. Figure 4a shows that the 95th percentile of the absolute values of the anatomic deviations measured by the 8 parameters, ranged from 7 to 11 mm for interfractional variations (median 8.5 mm) and form 5 to 9 mm (median 5.5 mm) for intrafractional variations. Despite the occurring anatomic variations, the clinical used margins of 5 mm were sufficient to meet the dosimetric requirement of an accumulated EUD_CTV_ > 95% for all adaptive treatment phases for different patients with 3 or more dose fractions by DIR-based dose accumulation. The median EUD_CTV_ was 101.7 (95.7–103.5%) for the accumulated scheduled plans and 102.7% (99.6–102.7%) for the accumulated adaptive plans. The above shown differences in the intra- and interfractional anatomic deformations gives a quantitative impression of the potential of adaptive radiotherapy to reduce PTV-margins in further studies.

In addition, we looked at the minimum PTV-margin at which the EUD_CTV_ for the accumulated plans were > 95% in all of the 8 treatment phases (with ≥ 3 fractions) and this was 5 mm for the scheduled plans, and 3 mm for the adaptive plans. At 2 mm PTV margins, two treatment phases had EUD_CTV_ values < 95% in the scheduled plan (79.0% and 82.8%). Table 2 summarises minimum, maximum, mean and median EUD_CTV_ over all 8 treatment phases in comparison for adaptive and scheduled plans.

**Table 2:** Accumulated dose distributions of EUD_CTV_ values for all 8 treatment phases with at least 3 dose fractions with the adaptive and scheduled plans in dependence on the PTV margins between 1 and 10 mm, respectively

	Adaptive plan				Scheduled plan			
	Minimum [%]	Maximum [%]	Mean [%]	Median [%]	Minimum [%]	Maximum [%]	Mean [%]	Median [%]
1 mm	83.2	102.5	98.9	101.4	71.9	102.8	93.7	98.0
2 mm	91.6	102.7	100.5	101.8	79.0	103.2	95.7	99.8
3 mm	99.1	102.8	101.6	102.0	86.0	103.5	99.9	101.8
4 mm	100.2	102.8	102.0	102.2	92.7	103.6	101.2	102.2
5 mm	100.2	104.1	102.1	102.2	98.3	103.7	102.0	102.2
6 mm	100.2	104.7	102.2	102.1	101.2	103.9	102.5	102.2
7 mm	100.2	105.1	102.1	102.0	101.1	104.1	102.6	102.4
8 mm	100.2	105.3	102.1	101.9	100.9	104.3	102.6	102.5
9 mm	100.3	105.4	102.0	101.9	100.8	104.5	102.5	102.5
10 mm	100.3	105.5	102.0	101.8	100.6	104.6	102.5	102.5

The distributions were characterized by the minimum, maximum, mean and median value and the range. All EUD_CTV_ values were normalized to the prescribed dose

**Fig. 5:**
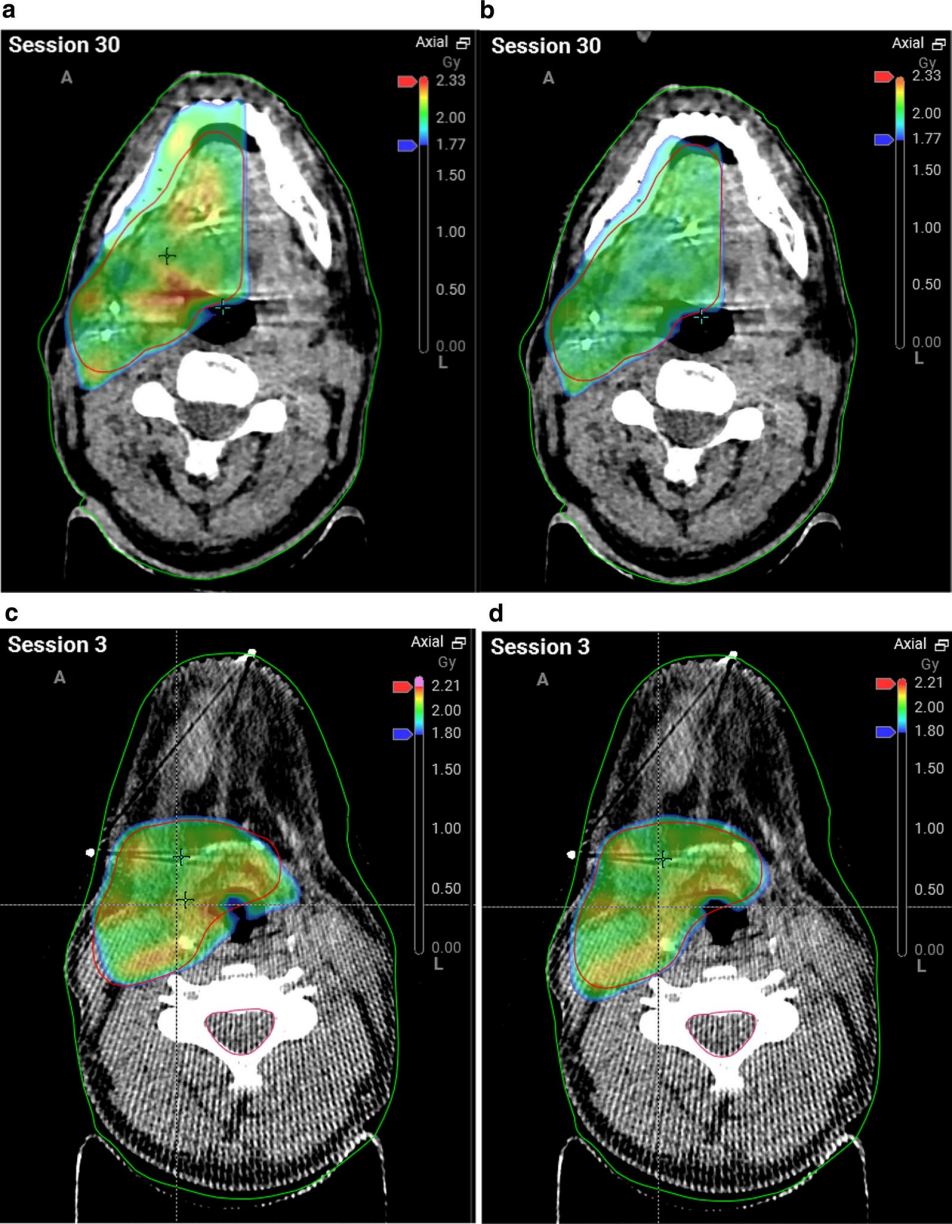
**a** Left: scheduled plan of a patient with oropharyngeal tumor of the right lateral border with less sparing of tongue, mandible and oral cavity, fraction 30, boost course, **b** right: adaptive plan of same patient with oropharyngeal tumor of the right lateral border with higher dose gradients to critical tissues and organs at risk, fraction 30, boost course, **c** left: scheduled plan in axial view of a patient undergoing the boost course, with less sparing of posterior pharyngeal wall. larynx and vocal cords, **d** right: adaptive plan of same patient in axial view with higher dose gradients to critical tissues, particularly to the posterior pharyngeal wall and larynx

In some patients, proper dose guidance was important for selected organs such as the oral cavity or mandible. Here it was possible to locally further improve dose gradients and optimize dose distribution to critical tissues (Fig. 5a–d).

## Discussion

It is important to distinguish between offline, online and hybrid workflows, as they represent fundamentally different forms of ART, but are nevertheless often referred to by the term ART [40]. This study is the first presenting data about kV-CBCT-based online adaptive radiation therapy in patients with tumors in the head and neck area from a prospective registry. We could show that patients with tumors of the oropharynx, particularly of the base of the tongue, and the mobile tongue may benefit from online adaptive radiation therapy, which is after all challenging in many ways. Adaptive plans show mainly a superior CTV coverage at a lower effective dose to the parotids and the larynx. Meanwhile, there was no significant difference in EUD for the spinal canal and the plexus brachialis, probably because the primary focus of IGRT was laid upon reproducible positioning of the vertebrae. There exist different modalities of 3-dimensional imaging, which allow adaptive radiation therapy by calculating the spatial dose, kV-CBCT, MV-CBCT or MR-guided [25], each of which has its limitations and challenges. An alternative to CBCT-based ART is MR-guided online adaptive radiotherapy (MRgART). The preliminary results of MR-guided radiotherapy of patients with tumors in the head and neck area and weekly plan adaptation are promising [41], but further studies are necessary to evaluate its clinical superiority. Van Timmeren et al. demonstrated significant changes in salivary gland volumes and position following daily MR guidance and weekly plan adaptation [41]. Likewise. Mulder et al. [42] reviewed clinical trials that had been started to evaluate the potential of offline adaptive radiotherapy with an MRI-linac to reduce normal tissue toxicity. At present, a few prospective randomized trials for ART in head and neck cancer are conducted, predominately adapting the treatment plan once a week, in order to evaluate the efficacy of ART in different clinical settings [e.g. NCT04883281, NCT01874587, NCT04901234, NCT04172753, NCT03972072, https://www.clinicaltrials.gov/]. Preceding, retrospective dosimetric studies report a beneficial dose distribution of ART compared to classical IGRT [14–17, 23, 25, 43]. A single prospective, randomized phase 3-trial published so far on weekly, offline plan adaptation showed a rather sober picture without a clear clinical benefit of offline ART compared to standard IGRT for the primary endpoint, sparing the parotid glands [24]. The randomized ARTIX phase III trial of definitive adaptive radiotherapy and adjuvant treatment of patients with locally advanced squamous cell carcinoma of the oropharynx showed no benefit of weekly offline adaptive radiotherapy on the primary endpoint of xerostomia compared to standard non-adaptive radiotherapy [24]. Weekly offline, adaptive radiotherapy is able to correct systematic interfractional anatomical changes, e.g. due to tumor shrinkage or weight loss. In the ARTIX trial, no dosimetric advantage of weekly dose adjustment in terms of a reduction of the mean dose to the parotid glands could be demonstrated, a prerequisite for a causal effect of dose adjustment on side effects. Likewise, a uniform PTV margin of 5 mm was applied in both arms. Nonetheless, the authors believe that adaptive radiotherapy could be used to spare other organs at risk, such as pharyngeal constrictors, which clearly could improve quality of life [24]. The pharyngeal constrictors play a key role in dysphagia-optimised intensity-modulated radiotherapy, which is considered as new standard of care for patients receiving radiotherapy for pharyngeal cancers [44]. The DARS trial for patients with oropharyngeal or hypopharyngeal cancer, which had a moderate size with 112 randomized patients [44], has recently shown that dose sparing of normal tissue can lead to an assessable benefit on a functional endpoint. In the experimental arm of this study, dose constraints were applied to the pharyngeal muscles and an improvement in swallowing function was observed after 12 months. For dysphagia-optimised intensity-modulated radiotherapy (DO-IMRT) a mandatory mean dose constraint of 50 Gy is reported for the volume of the superior and middle pharyngeal constrictor muscle or inferior pharyngeal constrictor muscle lying outside the high-dose target volume [44]. According to the randomized DARS phase III-trial, DO-IMRT improved considerably patient-reported swallowing function [44]. Furthermore, online adaptive radiation therapy may add to dose sparing not only of the constrictor muscles, but also of the oral cavity and tongue and, thus, to an improved quality of life, as mean radiation dose of oral cavity is reported to be closely linked to dysgeusia and dysphagia [45]. Castelli et al. discuss the limitations of once weekly offline adaptation and point out that recent software advancements allow real-time adaptive radiotherapy adapting the treatment to the daily shape of the patients by means of onboard CBCT or magnetic resonance imaging [24]. Contrary to weekly, offline plan adaptation, online onboard ART allows compensating instantly interfractional deviations and at the same time reducing PTV-margins, as well as optimizing mean and maximum doses to organs at risk. In the present study, an online adaptive radiotherapy capable of adapting to random and systematic interfractional anatomic deviations was applied and analyzed. Our results confirm that it is possible to improve the dose coverage of the CTV by simultaneously limiting dose to organs at risk in the head and neck area by online onboard ART. As shown in this study, online adaptive radiotherapy has the potential to reduce the PTV margins in tongue tumors and, thus, reduce the dose to the surrounding normal tissues. Therefore, the hypothesis that online adaptive radiotherapy can help to protect organs at risk in the area of the tongue such as the pharyngeal muscles, the oral cavity or the larynx is supported by the present study, but the clinical benefit of online adaptive radiotherapy must be demonstrated in prospective comparative studies.

According to Bruijnen et al. [46] the maximum intrafractional tumor motion in the head and neck area during resting is small and on average 2.8 mm in the superior–inferior direction and 2.1 mm in the anterior–posterior direction, when the patients do not swallow. However, in some individuals deviation may be even greater than 10 mm [46]. Motion during swallowing, which is inevitable during a treatment session, may lead to even larger movements. Using MR-CINE imaging Weiss et al. found that in the head and neck necessary margins to account for anterior/ posterior/ superior/ inferior tumor motion for oropharyngeal and laryngeal/ hypopharyngeal cancers were 4.1/4.4/5.0/6.2 mm and 4.9/4.3/6.7/7.7 mm [47]. Anticipating motion in advance is one important step how to handle the issue of deformation in the head and neck area. However, if one wants to account for all possible motion pathways, this will inevitably lead to large PTV margins in an area that is sensitive and where many critical organs are at risk in a confined space. The present study shows that margins covering 95% of anatomical deformations are not necessary. The 95th percentile of interfractional anatomic deviations measured by the 8 anatomic landmarks ranged from 7 to 11 mm, while the EUD_CTV_ for the accumulated dose distribution with IGRT stayed above 95% for all treatment phases with > 3 dose fractions using PTV margins of 5 mm.

Sparing normal tissue is an important goal in radiotherapy, especially in the head and neck area, as this includes many delicate structures that require special attention and care. Previous studies report that normal tissue toxicity could be underestimated in a curative setting for tumors in the head and neck area [10, 48].

The ART modus allows compensation for the loss of body weight and interfractional variations of the position of air/soft tissue interfaces by re-calculating the new dose to the anatomy of the day. As a result, the dose homogeneity can be considerably improved in the adaptive plan compared with the scheduled one as has been shown in the present study.

Previous studies support an early evaluation of the value of ART during the course of treatment [27, 40]. Gan et al. [13] propose a strategy to select patients for ART based on an observed NTCP increase of the delivered dose to organs at risk during the first two weeks of radiotherapy with IGRT. Developments to further automatize the unsupervised contouring of normal tissues and the dosimetric analysis of the dose distributions in normal tissues as important steps of ART will allow more selective use of ART to bridge the gap between resources and clinical practice. However, at present, despite artificial intelligence online onboard examination was performed in 100% by expert radiation oncologists and additionally for all adaptive-applied plans by supervision of a medical physicist. Expertise is needed at every step of the adaptive radiation therapy, checking contours of organs at risk, of segmented targets and technical quality parameters of adaptive and scheduled plans. The second CBCT for final verification combined with fast treatment delivery allows an intra-fraction monitoring. Thus, despite the aforementioned drawbacks, the system has some distinct advantages over traditional radiotherapy, which further enhances modern radiotherapy delivery. These include the perception of anatomical changes due to high image quality, instant onboard dose adjustment before each irradiation fraction, 1–3 mm margin concepts where necessary, particularly for critical organs at risk or in the case of re-irradiation, and an online onboard monitoring of adaptive therapy. Anatomical deviations tend to become also larger intrafractionally with adaptation time. Nonetheless, in this study greater anatomic mobility of anatomic landmarks around the tongue and base of the tongue was demonstrated interfractionally than intrafractionally for all examined landmarks. This indicates the potential of ART to reduce PTV margins around the CTV. The appropriate use of ART in the head and neck area for which radiotherapy is indicated is a field of further investigations. Poor EUD_CTV_ values by the scheduled plan are identified as a prerequisite for adaptive RT benefit. Thus, the clinical benefit for the use of the ART will depend on the patient and can be detected during the course of treatment by close monitoring of the accumulated dose distribution for normal tissues and the tumor.

A limitation of the present study is that not all, but only selected patients were treated with the ART mode under the discretion of the treating physician. On the prefractional CBCT1 a clear benefit of the adaptive plans was found in comparison to the scheduled plans, but adaptation takes time and it is important to assess intrafractional stability of the anatomy. A recalculation of the dose distribution on the 2nd CBCT could probably better estimate CTV coverage and the exposure of organs at risk and is work in progress. Online onboard kV-CBCT-based adaptation time in the present study took 33 min compared to adaptation time at MR-linacs for which an adaptation time of > 45 min is reported [49, 50]. Intrafractional deviations of anatomic landmarks, associated with deformations of the tongue were smaller than interfractional in this study. Therefore, prerequisites of the persistence of a gain of the adaptive plan throughout the fraction at given margins is fulfilled especially in tumors of the tongue and tongue base representing the highly mobile part of the oral cavity and oropharynx.

## Conclusions

The mobile tongue and tongue base showed considerable interfractional variations. While PTV- margins of 5 mm were sufficient for IGRT, ART showed the potential of decreasing PTV margins and spare dose to the organs at risk.

### Supplementary Information

**Additional file 1.** Supplementary file of inter- and intrafractional deviations of evaluated landmarks.

## Data Availability

The datasets used and/or analysed during the current study are available from the corresponding author on reasonable request.

## Abbreviations

ART: Adaptive radiation therapy
CBCT: Cone beam computed tomography
CTV: Clinical target volume
D_min_: Dose minimum or minima *(singular and plural)*
D_max_: Dose maximum or maxima *(singular and plural)*
DIR: Deformable image registration
EUD: Equivalent uniform dose
EUDaCTV: EUD within the CTV in the adaptive plan
EUDsCTV: EUD within the CTV in the scheduled plan
GTV: Gross target volume
IGRT: Image-guided radiotherapy
IMRT: Intensity modulated radiotherapy
MRgART: MR-guided online adaptive radiotherapy
OAR: Organs at risk
PTV: Planning target volume
VMAT: Volumetric modulated arc therapy
