# Supplementary material for: Prospects for online adaptive radiation therapy (ART) for head and neck cancer
Source: Radiat Oncol. 2024 Jan 8;19:4. doi: 10.1186/s13014-023-02390-6 (PMC10775598; doi:10.1186/s13014-023-02390-6)
Supplement: Supplementary file 1 — Additional file 1. Supplementary file of inter- and intrafractional deviations of evaluated landmarks. [file 13014_2023_2390_MOESM1_ESM.docx]

**Supplement**

**Supplementary material and methods**


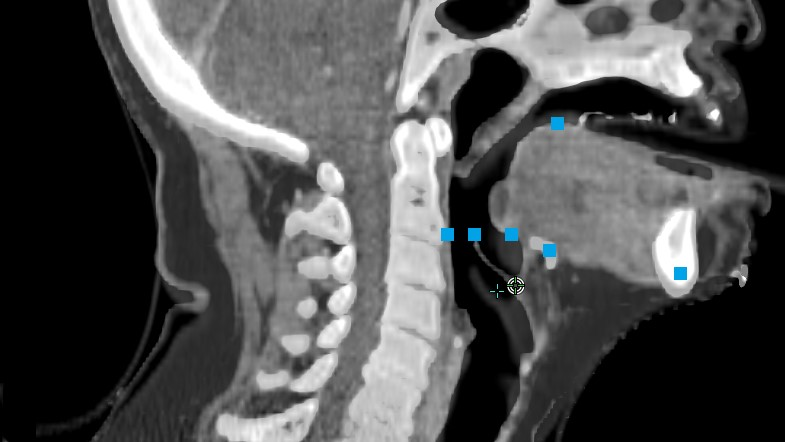

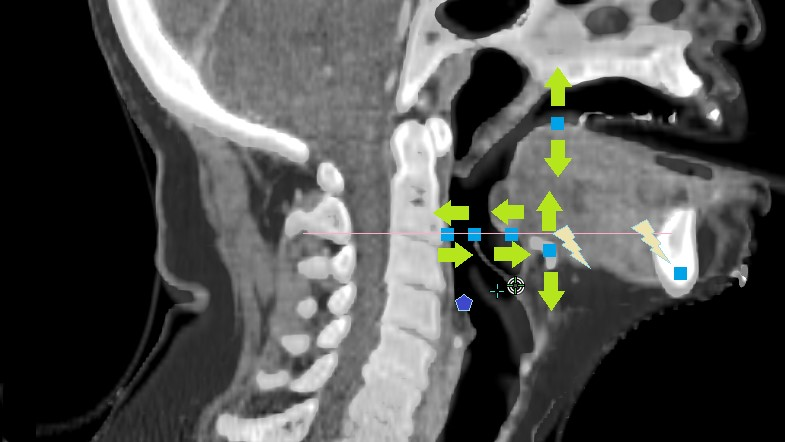


| **Supplementary Figure 1** | Inter- and intrafractional deformations evaluated by measuring specific points at predefined 8 landmarks (highlighted with red coloured stars) on the sagittal plane in the midline of the planning-CT, CBCT1 and CBCT2: (a) anterior/ posterior deviation of posterior pharyngeal wall at the tip of the epiglottis ; (b) maximum anterior/ posterior deviation of posterior pharyngeal wall ; (c) maximum deviation of mandible ;  (d) maximum craniocaudal deviation of os hyoideum ; (e) overall maximum deviation of os hyoideum ; (f) maximum anterior/ posterior deviation of tongue base ;(g) maximum craniocaudal deviation of tongue back ; and (h) anterior/ posterior deviation of tongue base at the tip of the epiglottis |
| --- | --- |

**Supplementary results**

When comparing other important CTV dose metrics of the adaptive applied fractions, it becomes obvious that D_99%_CTV_ and D_min_CTV_ differ significantly between adaptive and scheduled plans (*p* = 0.012; *p* = 0.020).
